# Multifunctional layered magnetic composites

**DOI:** 10.3762/bjnano.6.13

**Published:** 2015-01-12

**Authors:** Maria Siglreitmeier, Baohu Wu, Tina Kollmann, Martin Neubauer, Gergely Nagy, Dietmar Schwahn, Vitaliy Pipich, Damien Faivre, Dirk Zahn, Andreas Fery, Helmut Cölfen

**Affiliations:** 1 Department of Chemistry, Physical Chemistry, University of Konstanz, Universitätsstraße 10, 78457 Konstanz, Germany; 2 Jülich Centre for Neutron Science JCNS-MLZ, Outstation at MLZ, Forschungszentrum Jülich, Lichtenbergstraße 1, 85748 Garching, Germany; 3 Theoretical Chemistry, University of Erlangen-Nürnberg, Nägelsbachstraße 25, 91052 Erlangen, Germany; 4 Physical Chemistry II, University of Bayreuth, Universitätsstraße 30, 95447 Bayreuth, Germany; 5 Laboratory for Neutron Scattering, Paul Scherrer Institute, 5232 Villigen PSI, Switzerland; 6 Technische Universität München, Forschungs-Neutronenquelle Heinz Maier-Leibnitz (FRM II), 85748 Garching, Germany; 7 Department of Biomaterials, Max Planck Institute of Colloids & Interfaces Science Park Golm, 14424 Potsdam, Germany

**Keywords:** bio-inspired mineralization, biomineralization, chitin, ferrogel, hybrid materials, magnetite, nacre

## Abstract

A fabrication method of a multifunctional hybrid material is achieved by using the insoluble organic nacre matrix of the *Haliotis laevigata* shell infiltrated with gelatin as a confined reaction environment. Inside this organic scaffold magnetite nanoparticles (MNPs) are synthesized. The amount of MNPs can be controlled through the synthesis protocol therefore mineral loadings starting from 15 wt % up to 65 wt % can be realized. The demineralized organic nacre matrix is characterized by small-angle and very-small-angle neutron scattering (SANS and VSANS) showing an unchanged organic matrix structure after demineralization compared to the original mineralized nacre reference. Light microscopy and confocal laser scanning microscopy studies of stained samples show the presence of insoluble proteins at the chitin surface but not between the chitin layers. Successful and homogeneous gelatin infiltration in between the chitin layers can be shown. The hybrid material is characterized by TEM and shows a layered structure filled with MNPs with a size of around 10 nm. Magnetic analysis of the material demonstrates superparamagnetic behavior as characteristic for the particle size. Simulation studies show the potential of collagen and chitin to act as nucleators, where there is a slight preference of chitin over collagen as a nucleator for magnetite. Colloidal-probe AFM measurements demonstrate that introduction of a ferrogel into the chitin matrix leads to a certain increase in the stiffness of the composite material.

## Introduction

Biominerals, which are organic–inorganic hybrids and highly sophisticated materials with optimal assimilated properties, have evolved in nature. The mechanisms of biomineral formation are still far from being understood and there is currently large research activity from groups of different expertise. Most biominerals are hierarchically structured, which consequently adds favorable physical properties such as hardness and fracture resistance to the material. An intriguing and much investigated material is nacre which is the inner protecting layer of some marine sea shells. It is well-known for its beautiful iridescence but also for the outstanding mechanical properties. Nacre has a layered structure of aragonite platelets and an organic matrix mainly consisting of β-chitin covered with proteins [1]. This hybrid structure makes nacre 3000 times more fracture resistant as compared to aragonite which makes up ca. 95 wt % of this structure [2]. The reason for this is that crack propagation is hindered by the soft chitin layers that get disrupted before the crack can propagate further. In addition, the platelets are glued to the organic matrix by elastic proteins that also have sacrificial physical bonds [3]. Another amazing biomineral are chiton teeth, which are actually the hardest known biomineral [4]. Chitons scratch algae from rocks, which requires wear-resistant teeth. The animal maintains this ability by synthesizing rows of teeth and each time, a tooth is worn out, the next tooth in the row will be used. A reason for the mechanical wear resistance of the teeth is the presence of different iron oxide mineral phases incorporated into a protein–polysaccharide matrix. Especially, magnetite nanoparticles that are present in large amounts (ca. 70 wt %) at the tooth cap, covering the cutting surface, are responsible for the outstanding mechanical performance [5].

There are many approaches to produce an organic–inorganic hybrid material inspired by the structure of nacre [6–16]. But the fundamental knowledge of the underlying mechanisms as well as theoretical explanations were, so far, only provided for rare examples. One of the reasons is that many of the biomineralization mechanisms are still not fully understood due to their complexity. Recent work underlines the importance of amorphous precursor phases [17] and also nonclassical crystallization mechanisms in biomineralization [18–19].

In this manuscript we report a synthesis method to combine the favorable properties of two biominerals in one and the same material and, thus, to create a multifunctional hybrid material. We claim that this bioinspired material could find potential application in various fields. In general, it could be very interesting for the field of abrasive and fracture resistant materials that are found in hard coatings or in the field of construction. We used the organic nacre matrix of the shell *Haliotis laevigata*, which is insoluble in acetic acid, as a confined reaction environment. Within this organic matrix we infiltrated gelatin to mimic the silk gel precursor inside the chitin nacre scaffold [3]. Inside this organic gelatin matrix we synthesize magnetite nanoparticles to form a highly mineralized organic–inorganic hybrid body. The resulting material should mimic the fracture resistance of nacre and the hardness and abrasion resistance of the chiton teeth.

## Results and Discussion

### Synthetic concept

**Figure 1:**
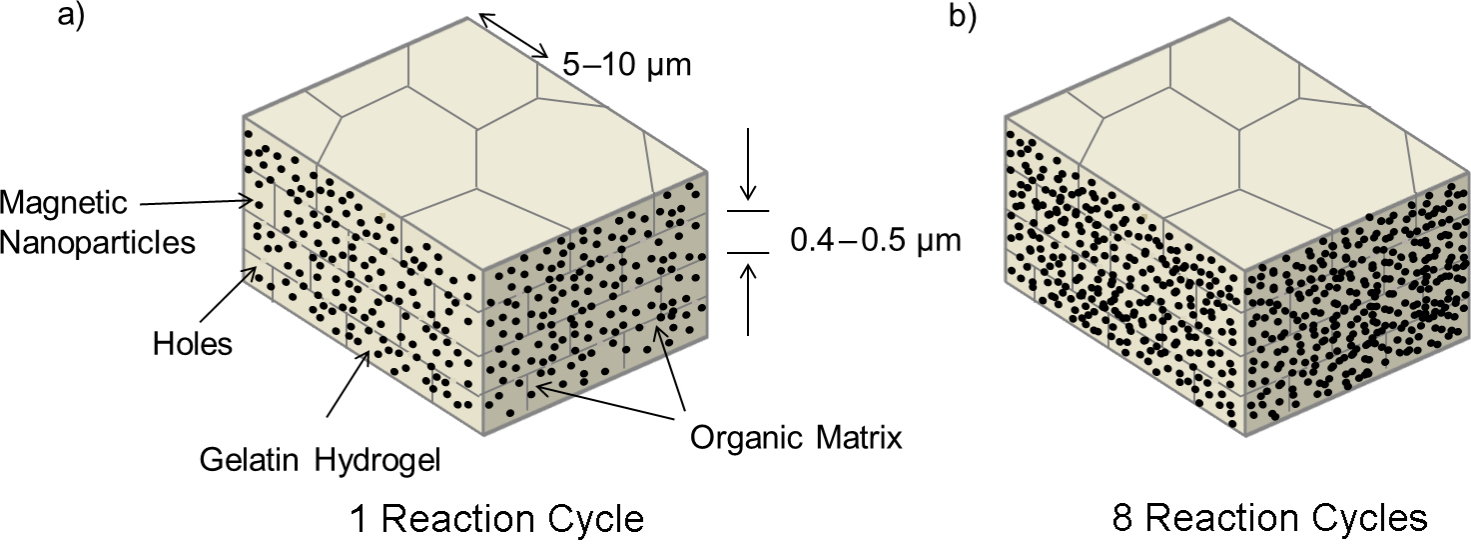
Magnetite formation inside a gelatin gel matrix (grey) that is placed inside the chitin scaffold of demineralized nacre (dark grey lines). Panel a) symbolizes the stage of mineralization after one reaction cycle, panel b) represents repeated mineralization cycles as demonstrated by the progress of mineralization shown by the increase of the magnetite nanoparticle number. At zero time, only the gel matrix is present.

It is the aim to synthesize a material of larger dimensions by developing a multifunctional biomimetic composite structure, which combines properties of two biominerals in one and the same material, namely nacre and chiton teeth. To reach this goal we follow the key synthesis principles presented in Figure 1. The starting material is an original demineralized nacre matrix that is infiltrated by a thermo reversible gelatin solution mimicking a gel precursor inside the chitin nacre scaffold. Within this gelatin matrix we synthesize magnetite nanoparticles to form a highly mineralized organic–inorganic hybrid body. This hybrid structure resembles the nacre aragonite platelets in size and shape. We repeated this reaction cycle up to eight times in order to enhance and thereby control the content of magnetite NPs inside the hybrid material. Systematic studies showed that after eight reaction cycles the upper limit of mineral load is reached and further repetition does not lead to an increase in the mineral content.

### Nacre organic matrix – SANS

**Figure 2:**
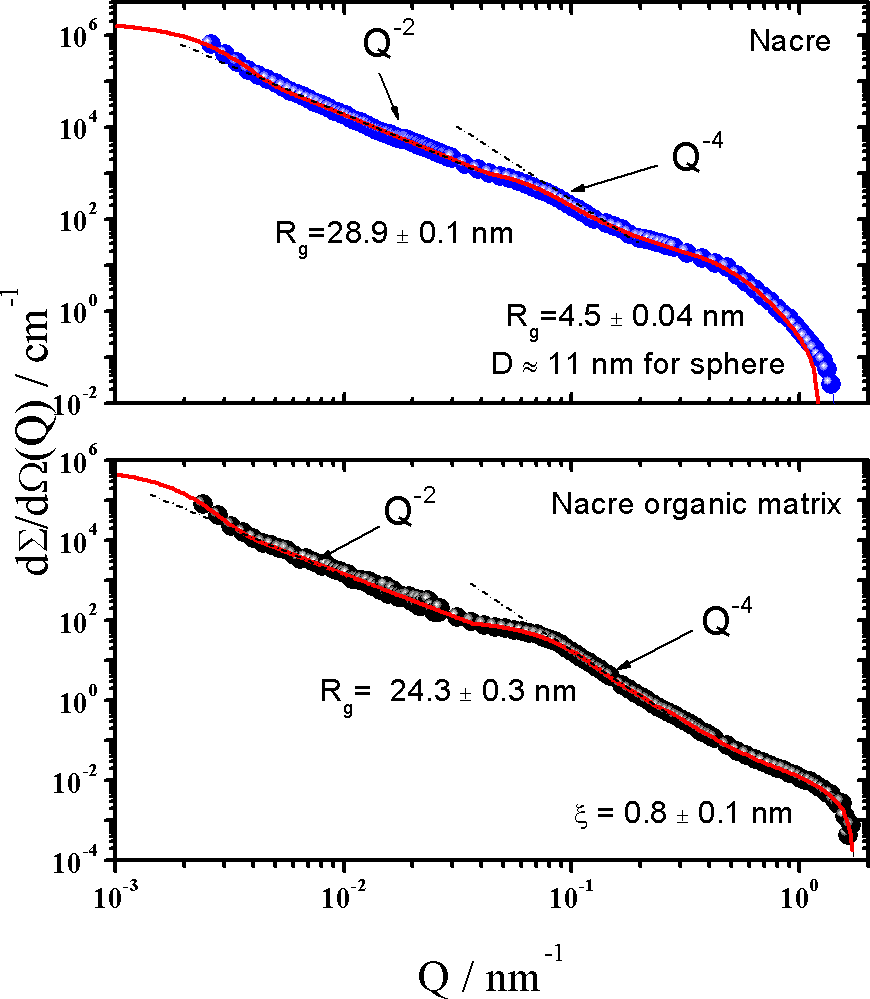
SANS macroscopic cross-section dΣ/dΩ versus scattering vector *Q* for a 1 mm thick piece of nacre in air and a demineralized nacre matrix in D_2_O (*T* = 20 °C). The neutron beam is parallel to the nacre/nacre organic matrix *c*-axis (perpendicular to the sample surface). At low *Q* (<0.02 nm^−1^) VSANS data are also presented after rescaling. The solid line represents a fit of the Beaucage equation [30] and correlation length model (*Q* > 0.03 nm^−1^) [31] (see Supporting Information File 1).

Nacre, an inorganic and organic composite natural material, is typically found as the inner shells of mollusks, and is referred to as mother-of-pearl. Its structure is a layered arrangement of pseudo-hexagonally shaped aragonite mesocrystals with a diameter of around 10–15 μm and a thickness of about 500 nm [3] (every platelet consists of polygonal CaCO_3_ nanograins with a size of 10–45 nm [20]). The aragonite mesocrystals are interspaced by an organic matrix which was identified as a β-chitin [21–23] core surrounded by protein layers that play an important role in the formation process of nacre [24–26]. The inorganic mesocrystals are connected by mineral bridges with a width ranging from 36–54 nm in between the neighboring lamellae. The mineral bridges represent the continuation of mineral growth along the vertical direction of the lamellar mesocrystals from a preceding layer of platelets [27–28]. The fraction of the organic matrix in nacre is only about 5 wt %, it plays an important role in the spatial control of mineralization, hierarchical structure and toughness enhancement [23,29]. Different techniques have been used to resolve the chemical and structural composition of the organic matrix. Small angle neutron scattering (SANS) is a non-destructive method to study the nacreous organic matrix without potential changes to the matrix, which might derive from the usage of staining media or dehydration. For comparison studies, the structure of the original nacre matrix (*Haliotis laevigata*) was analyzed as well. Figure 2 represents very-small (VSANS) and small (SANS) angle neutron scattering profiles of nacre (top) and its organic matrix (bottom) measured at two diffractometers for very small (VSANS) and conventional small angular scattering (SANS) in, respectively, *Q*-ranges from 10^−3^ to 2·10^−2^ nm^–1^ and from 10^−2^ to 3.5 nm^−1^. The absolute value of the scattering vector *Q* is related to the scattering angle θ and neutron wavelength λ according to Q = (4π/λ)·sin(θ/2). The neutron beam is parallel to the *c*-axis of the nacre or the organic matrix of the nacre (i.e., perpendicular to the sample surface, see Supporting Information File 1). Thus, nearly no information about the thickness of the lamellar platelets is found in the scattering curves. These measurements enable the determination of the hierarchical structures along the vertical direction of the lamellar platelets of the nacre and its organic matrix over a wide range of length scales from about 1 nm to 1 μm. The data in Figure 2 show several distinct *Q*-regimes that are described well by the solid line representing the best fit of the data using Beaucage’s expression [30] and a correlation model [31] (see Supporting Information File 1). For nacre, scattering from the aragonite mesocrystals is dominant in the *Q*-regime less than 0.02 nm^−1^ and is represented by a *Q*^−2^ power law with an amplitude of *P*_2_ = 1.8 cm^−1^·nm^−2^. This exponent implies a platelet-like structural characteristic with a plate diameter larger than 2 μm as evaluated from the radius of gyration, *R*_g_, assuming the form factor of a thin plate-like shape [32]. Above Q* 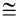 0.063 nm^−1^ the power law transforms into Q^−3^ and above 0.4 nm^−1^ to a Q^−4^ Porod behavior that yields an average size of the nanograins of about 10 nm as estimated from *D* ≡ 2π/Q^*^. The diameter of the nanograins is around 11 nm as evaluated from *R*_g_, assuming the form factor of a spherical shape, which is consistent with data reported in literature [29]. The scattering, which follows the Q^−2^ power law between 3·10^−2^ and 0.2 nm^−1^ shows the presence of a shoulder which might correspond to the mineral bridges with an average diameter that is estimated to be *D* ≈ 80 nm from *R*_g_ ≈ 28.9 nm.

The scattering profile of the nacre organic matrix (Figure 2, bottom) indicates the same platelet-like structure as for nacre as it shows the same power laws, however with an amplitude of *P*_2_ = 0.13 cm^−1^·nm^−2^, which is one order of magnitude smaller. This means that the demineralization has no significant influence on the original structure of the organic matrix. Above *Q* = 0.03 nm^−1^ a radius of gyration *R*_g_ of about 24.3 nm is determined, which might correspond to the mineral bridges. The diameters of the cross section of the bridges were estimated to be roughly D ≈ 68 nm from *R*_g_ ≈ 24.3 nm. This result is consistent with our TEM results. The size of the mineral bridge is much larger than the typical size of the gelatin molecule as determined from the correlation length ξ ≈ 15.9 ± 0.5 nm with SAXS (see Supporting Information File 1). This indicates that the molecular diffusion of gelatin into the organic chitin matrix through holes in the chitin layers, which originates from the former mineral bridges, is possible. Above *Q* = 0.5 nm^−1^ scattering from around 0.8 nm large particles appear, representing the scattering of the chitin chain. In summary, we can conclude that nacre is completely demineralized by our experimental procedure, which was also confirmed by TGA measurements, and that the structure of the demineralized nacre organic matrix has not significantly changed compared with the original nacre.

### Nacre organic matrix – Light microscopy and fluorescence microscopy

**Figure 3:**
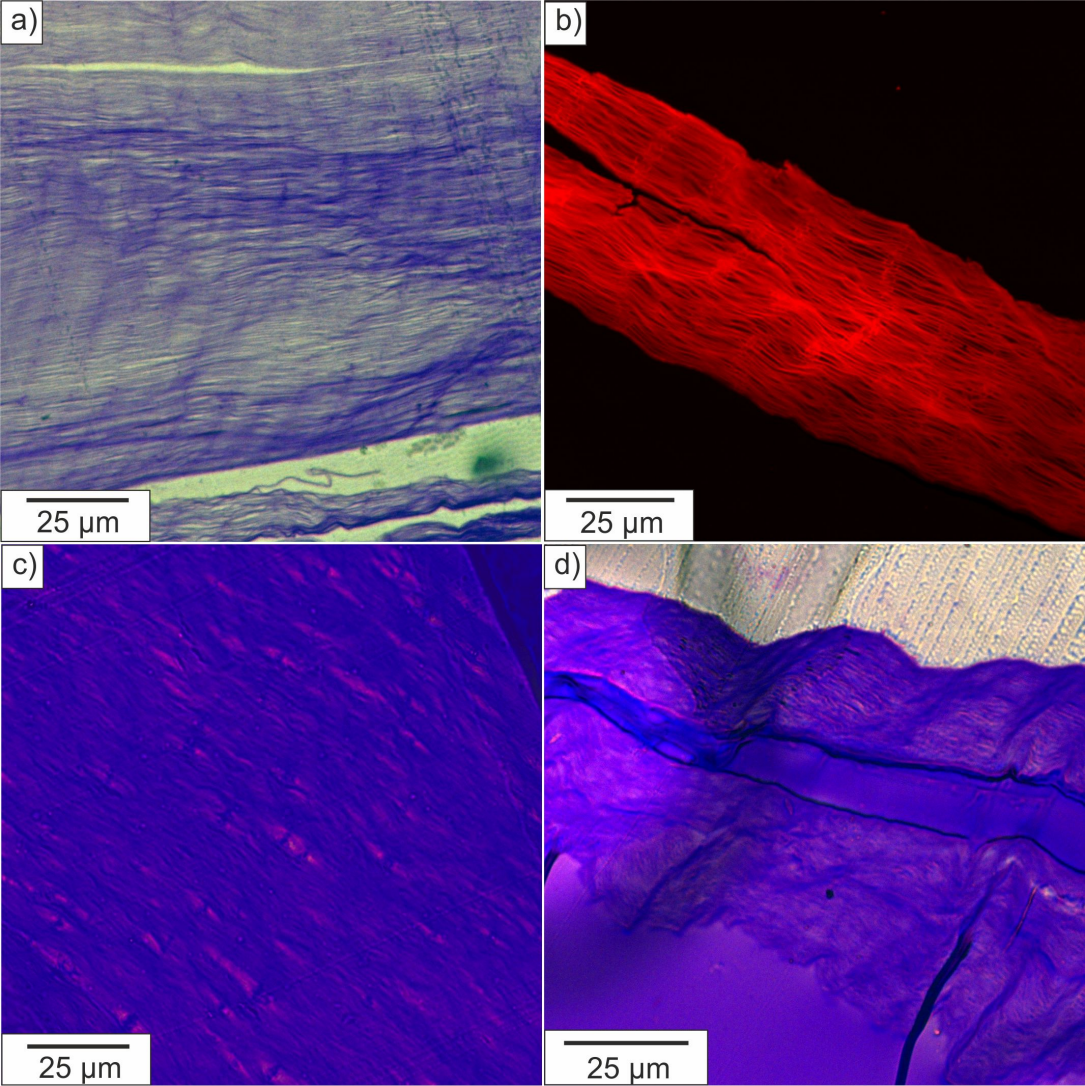
Light microscopy image of thin cuts of embedded and Coomassie stained samples. a) Demineralized nacre matrix, b) confocal laser scanning microscopy of embedded demineralized nacre matrix stained with rhodamine B ITC, c) and d) demineralized nacre matrix (blue) with infiltrated gelatin (purple).

**Figure 4:**
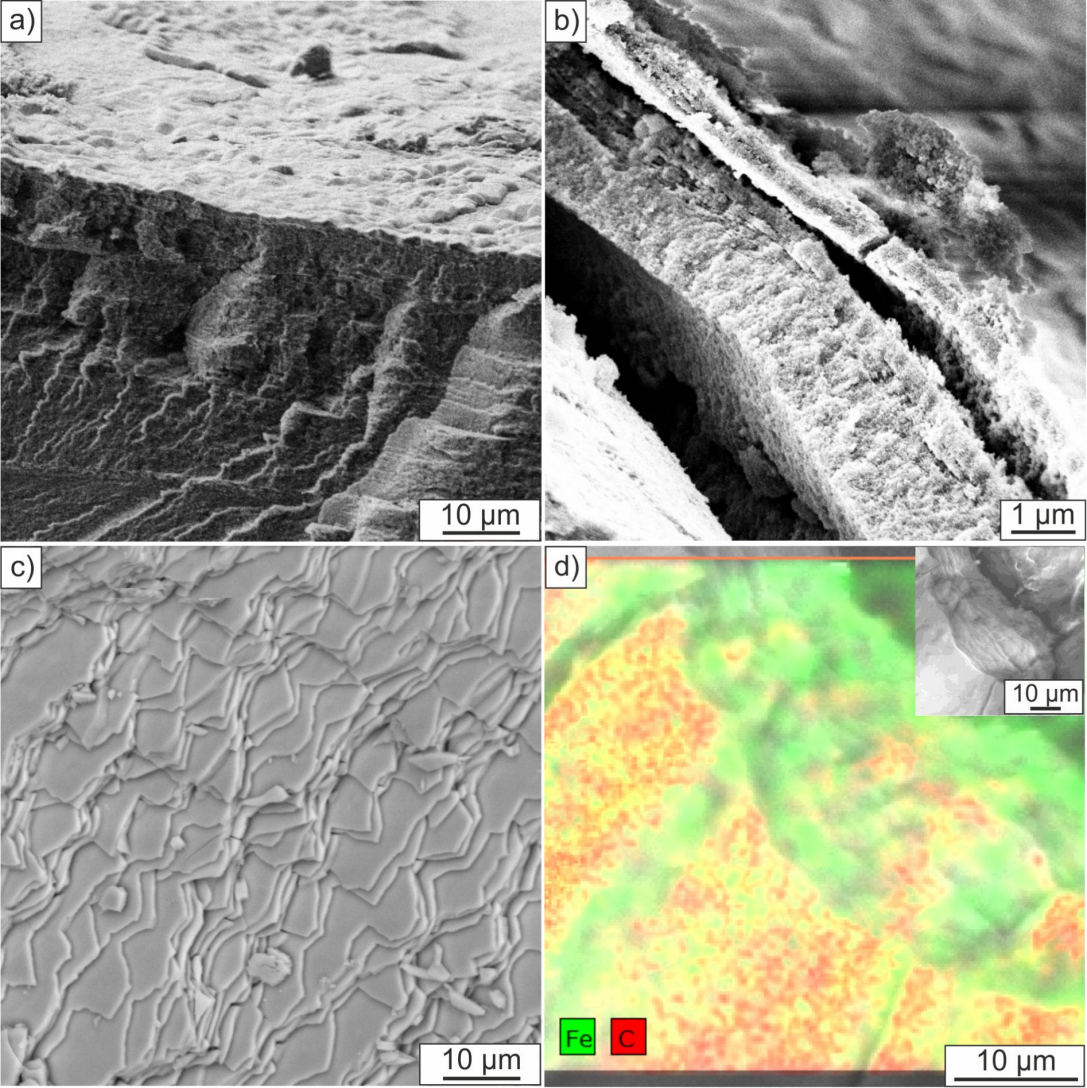
SEM micrographs of a) and b) fracture surfaces of artificial nacre and c) fracture surface of original nacre *Haliotis laevigata*. d) EDX mapping analysis of artificial nacre fracture surface.

**Figure 5:**
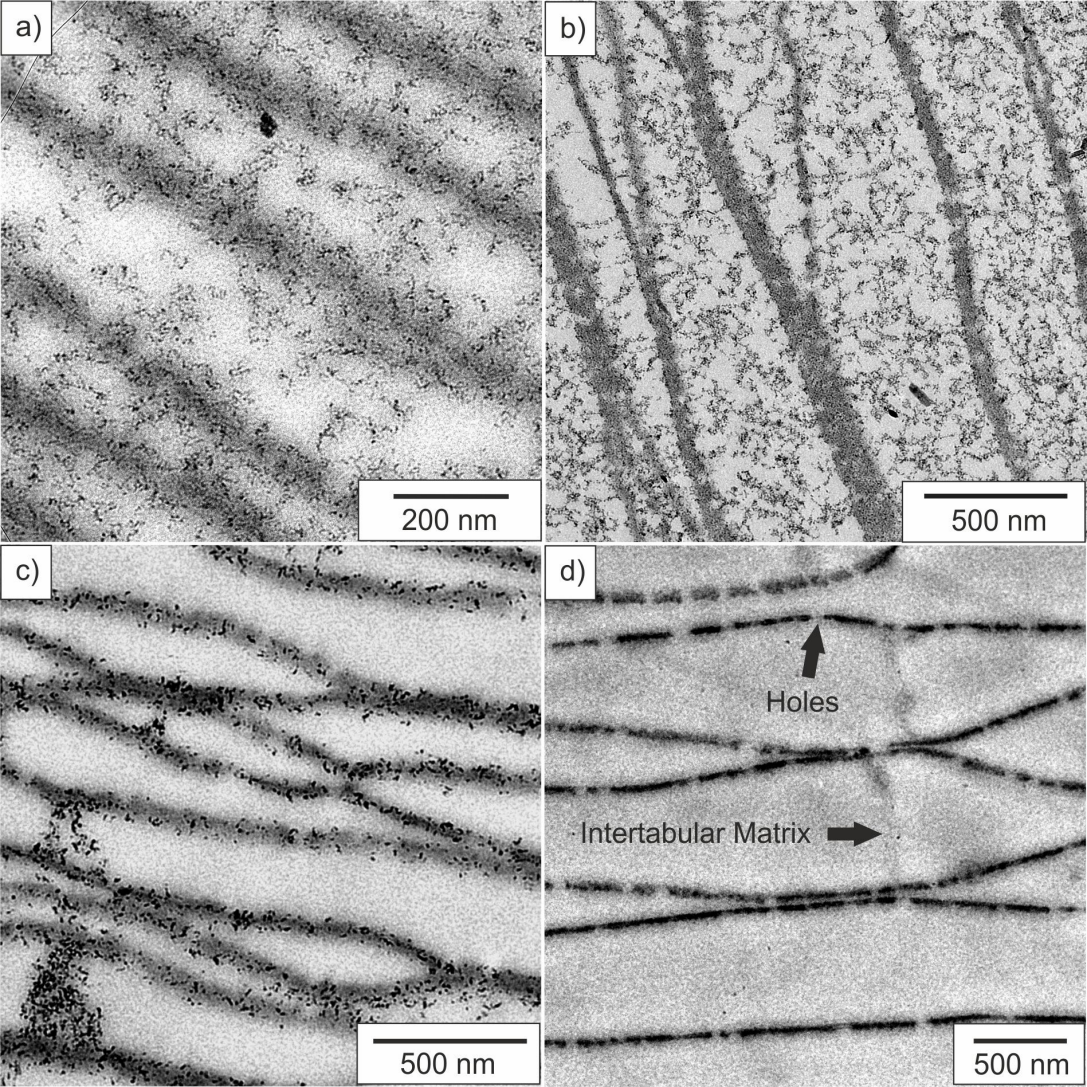
TEM micrographs of a) artificial nacre after one reaction cycle and b) after four reaction cycles, c) reference chitin–magnetite composite sample without gelatin, and d) completely demineralized matrix.

Original nacre (*Haliotis laevigata*) used for materials synthesis was analyzed by light microscopy and confocal fluorescence laser scanning microscopy (FCLSM) as can be seen in Figure 3. A freshly broken cross section of original nacre was analyzed by SEM (see below in Figure 4c) and clearly reveals the layered structure of aragonite tablets. The insoluble organic matrix can be seen in Figure 3 and in the transmission electron microscopy (TEM) image given below in Figure 5d. The embedded cross section of the demineralized chitin matrix shows that the matrix remains stable after demineralization and does not stick together. These results are in agreement with our findings from SANS and VSANS experiments, therefore we conclude that the demineralized nacre matrix can be used as a template for the synthesis of the composite material, which is in agreement with earlier work on nacre retrosynthesis [13]. The distance between the layers is around 250–500 nm (see below in Figure 5d), which is in part lower than that of the natural archetype (500 nm) due to a partial collapse of the demineralized matrix during preparation and handling. Figure 5d also illustrates vertical connections between the layers, these thin walls are the so-called “intertabular matrix” which has a stabilizing function [33]. The interruptions in the layers correspond to pores of around 50–70 nm thickness and act as mineral transport bridges during the formation of natural nacre, as also confirmed by SANS and VSANS experiments. In order to determine the arrangement of gelatin in between the insoluble organic nacre matrix layers a Coomassie stain is used. The light microscopy image in Figure 3a shows an embedded and thin cut section of demineralized nacre stained with Coomassie blue. The investigations clearly display a blue stain of the layered insoluble nacre structure as a result of a positive interaction of the insoluble proteins with Coomassie blue, whereas the space in between the layers does not show any significant stain. The same observation can be made by fluorescence confocal laser scanning microscopy (Figure 3b) for which the thin cuts have been stained with rhodamine B ITC. Also in these studies no staining of proteins in between the layers could be observed. Therefore we conclude that the insoluble matrix proteins are dominantly located directly at the β-chitin matrix and are not present in between the layers. Figure 3c and Figure 3d show an embedded sample of insoluble nacre matrix infiltrated with gelatin by a vacuum infiltration process. Staining of this sample illustrates not only blue stained chitin layers and insoluble matrix proteins but also colored areas in between the layers, indicating a filling of the matrix with gelatin. The interaction and positive stain of gelatin and Coomassie blue have been tested successfully in reference experiments (see Figure S3, Supporting Information File 1). For a better visualization of the stained areas in between the layers the light blue stained gelatin (see Supporting Information File 1) has been processed digitally. This means the green RGB channel of the images was exchanged by the red one to be able to better distinguish between the different matrix parts. As a result the stained gelatin parts appear purple in the image which makes it easier to differentiate between the blue chitin layers and the filling in between the layers. The purple area next to the matrix in Figure 3d represents excess of gelatin on the sample surface. These studies reveal that the chitin–gelatin composite can be used as a template for the mineralization of magnetite and therefore act as a building block for the formation of a multifunctional composite material. One key step for the formation of such a multifunctional hybrid material is the homogeneous infiltration of gelatin as the organic scaffold for mineralization inside the insoluble nacre matrix.

### General synthesis protocol and TEM/SEM studies

The synthesis of the multifunctional inorganic hybrid material is based on an already established three step protocol [34]. In the first step, the gelatin hydrogel is infiltrated into the demineralized nacre matrix through a vacuum infiltration process [35], in the second step this chitin–gelatin composite is introduced into a solution of ferrous (FeCl_2_ 0.1 M) and ferric ions (FeCl_3_ 0.2 M) in a molar ratio of 1:2. After complete diffusion of the ions inside the hydrogel template magnetite is precipitated in the third step by introducing the template in a base (NaOH 0.1 M). The magnetite nanoparticles are synthesized through a so-called co-precipitation method following the reaction:

[1]
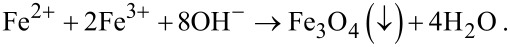


This procedure can be repeated several times in order to obtain the desired degree of mineralization. We already reported a similar synthesis protocol for gelatin-based magnetic hydrogels [34] and now transfer these synthesis principles into the insoluble organic nacre matrix.

The amount of magnetite nanoparticles formed inside the synthesized hybrid material was determined by thermogravimetric measurements. The initial and final degradation temperatures have been determined from the thermogram curves. The loading of the composite material with iron oxide nanoparticles varies from 15–65 wt % depending on the number of reaction cycles (see Figure S3, Supporting Information File 1). Scanning electron microscopy (SEM) examinations of the dried hybrid materials indicate a dense layered hierarchical structure (see Figure 4a), which is similar to natural nacre. The distribution of magnetite nanoparticles inside the hybrid material was determined with electron dispersive X-ray spectroscopy (EDX) (Figure 4d). The mapping of the elements shows that Fe and C are homogeneously distributed throughout the material surface whereas there is less C detected at the freshly broken cross section of the material. It can be clearly seen that the spaces in between the layers mainly give signals for Fe. With the performed studies we could not observe a mineral gradient throughout the matrix arising from the synthesis of the magnetic nanoparticles produced by a diffusion approach. Therefore we claim that after full completion of the synthesis the particles are equally present over the whole matrix.

In order to confirm this observation and to obtain information about the mineral nature in between the chitin sheets, TEM studies of embedded and microtome cut samples have been conducted (Figure 5). We note the presence of iron oxide nanoparticles homogeneously distributed in between the layers after one (Figure 5a) and four reaction cycles (Figure 5b). Moreover, it can also be seen that the number of particles after one reaction cycle is significantly lower than after four reaction cycles, which is in agreement with TGA studies of the hybrid materials. The studies show that the particles are in the size range of 10 ± 5 nm and do exist at the chitin surface as well as in between the chitin layers due to the presence of the carrying media gelatin. It is also worth to mention that besides the 10 nm sized particles also smaller particles in the size range of around 3 nm can be detected. Electron diffraction studies of these small particles show their amorphous nature, which leads to the conclusion that under the chosen synthesis conditions amorphous material or poorly crystallized ferrihydrite could be present. In this study we could not recognize a direct formation of magnetite through an amorphous or ferrihydrite precursor stage. However, the transformation of amorphous iron oxide species into magnetite was observed before and is also likely to happen in this synthesis set-up [36]. Reference experiments of the composite material without gelatin infiltration (Figure 5c) and repetition of four reaction cycles only show the presence of nanoparticles adsorbed on the chitin surface but not in between the layers. This material seems closer to the demineralized nacre matrix (Figure 5d) than to a multilayered composite material. Furthermore, the distance in between the layers for samples containing gelatin seems less collapsed than for samples without gelatin which results in a material closer in structure to that one of original nacre. Electron diffraction data taken from different areas in between the layers show the presence of polycrystalline nanoparticles with no preferred orientation (see Figure S5, Supporting Information File 1). The iron oxides magnetite and maghemite show very similar diffraction patterns and *d*-spacings, therefore it is not possible to differentiate these mineral phases with the used techniques. In summary these observations demonstrate that it is possible to successfully infiltrate a demineralized nacre matrix with gelatin and to form magnetite nanoparticles inside the gel matrix.

### SANS on magnetite formation in the gelatin–chitin composite

**Figure 6:**
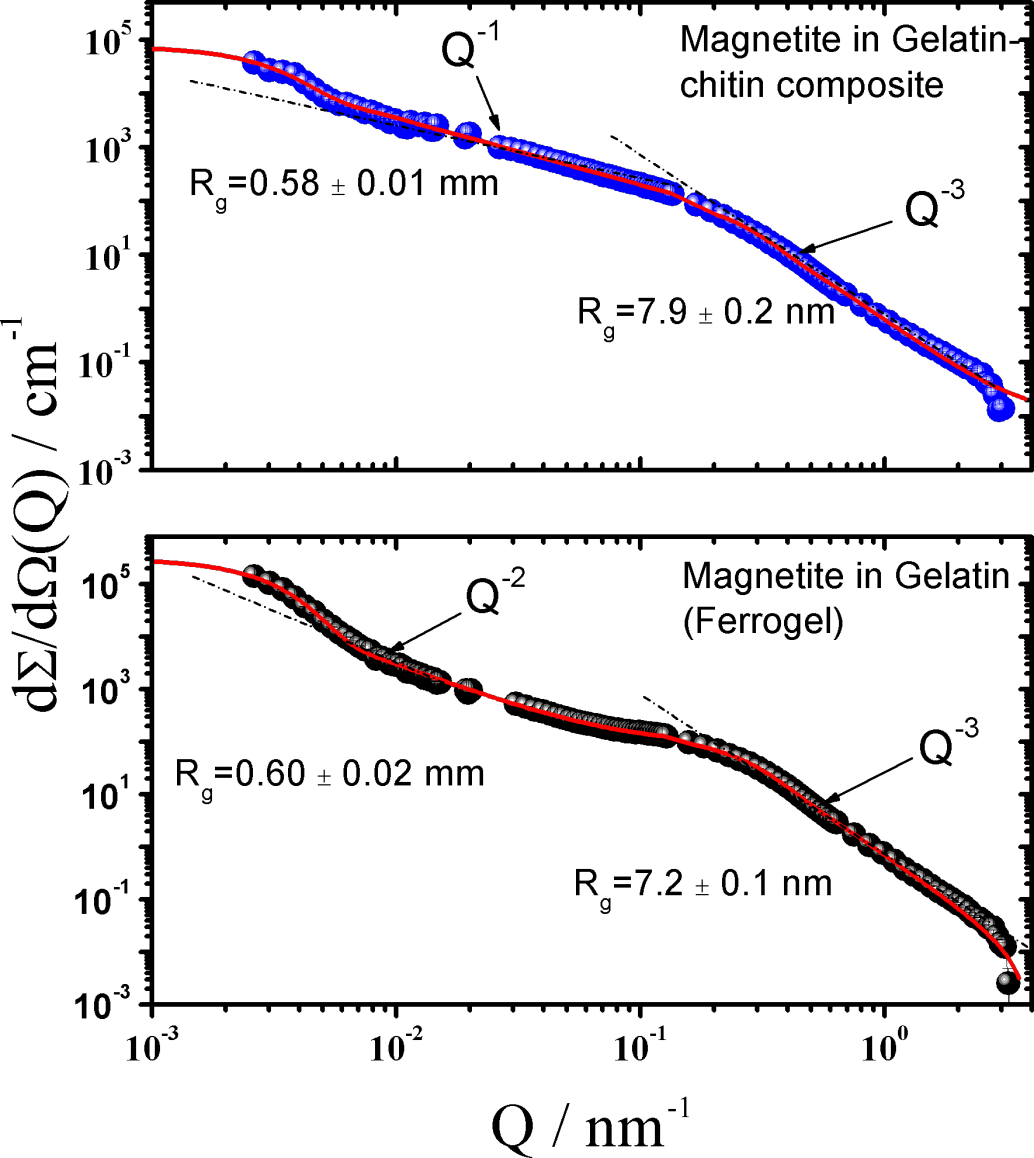
SANS and VSANS scattering patterns of magnetite in gelatin–chitin composite and of ferrogel in a mixed D_2_O/H_2_O solvent of 28 vol % D_2_O and 72 vol % H_2_O. The solid lines represent the fitting of the Beaucage expression [30].

The magnetite–gelatin–chitin structure was characterized by SANS contrast variation experiments, which is a beneficial method to obtain information about the inorganic components as well as the organic part. By using the matching point of gelatin (28 vol % D_2_O) only the inorganic particles are visible whereas the organic structure can be visualized working in pure D_2_O. This technique is a standard tool in various fields such as biomineralization [37–38]. Figure 6 demonstrates two SANS–VSANS scattering profiles of magnetite in a chitin–gelatin composite (top) and as a reference in a gel matrix (bottom). The structure of the ferrogel (the hybrid material without chitin) was investigated for comparison. The magnetite–gelatin–chitin sample shows a power law of *Q*^−1^ in the low-*Q* regime (<0.01 nm^–1^), which is approximately valid for linear structures and thereby indicates rod-like particles or chains of particles of about *R*_g_ = 0.58 μm. At larger *Q* (>0.1 nm^−1^) scattering is determined from individual magnetite nanoparticles of *R*_g_ 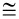 7.9 nm showing a Q^−3^ power law indicating a mass fractal structure (a structure containing branching and crosslinking to form a 3D network). The diameter *D* of the magnetite particles can be estimated to be *D* ≈ 20 nm (*R*_g_ = *D*/2.58) assuming a spherical shape. The scattering of magnetite in the gelatin matrix (ferrogel) qualitatively looks the same. Particles (or an assembly of particles) of about *R*_g_ = 0.6 μm with Q^−2^ power law, which is characteristic for chain-like clusters, are found at small *Q*. Individual magnetite particles become visible at larger *Q* showing a slightly smaller diameter of about *D* ≈ 18.5 nm (*R*_g_ = 7.2 nm). Thus, in the presence of nacre organic matrix, the fiber-like chitin structure helps with the formation of linearly aligned magnetite nanoparticles (pearl-necklace-like, power law of *Q*^−1^), while in the gelatin gel matrix without chitin, the nanoparticles exhibit a branch-like arrangement (power law of *Q*^−2^).

### Magnetization measurements

**Figure 7:**
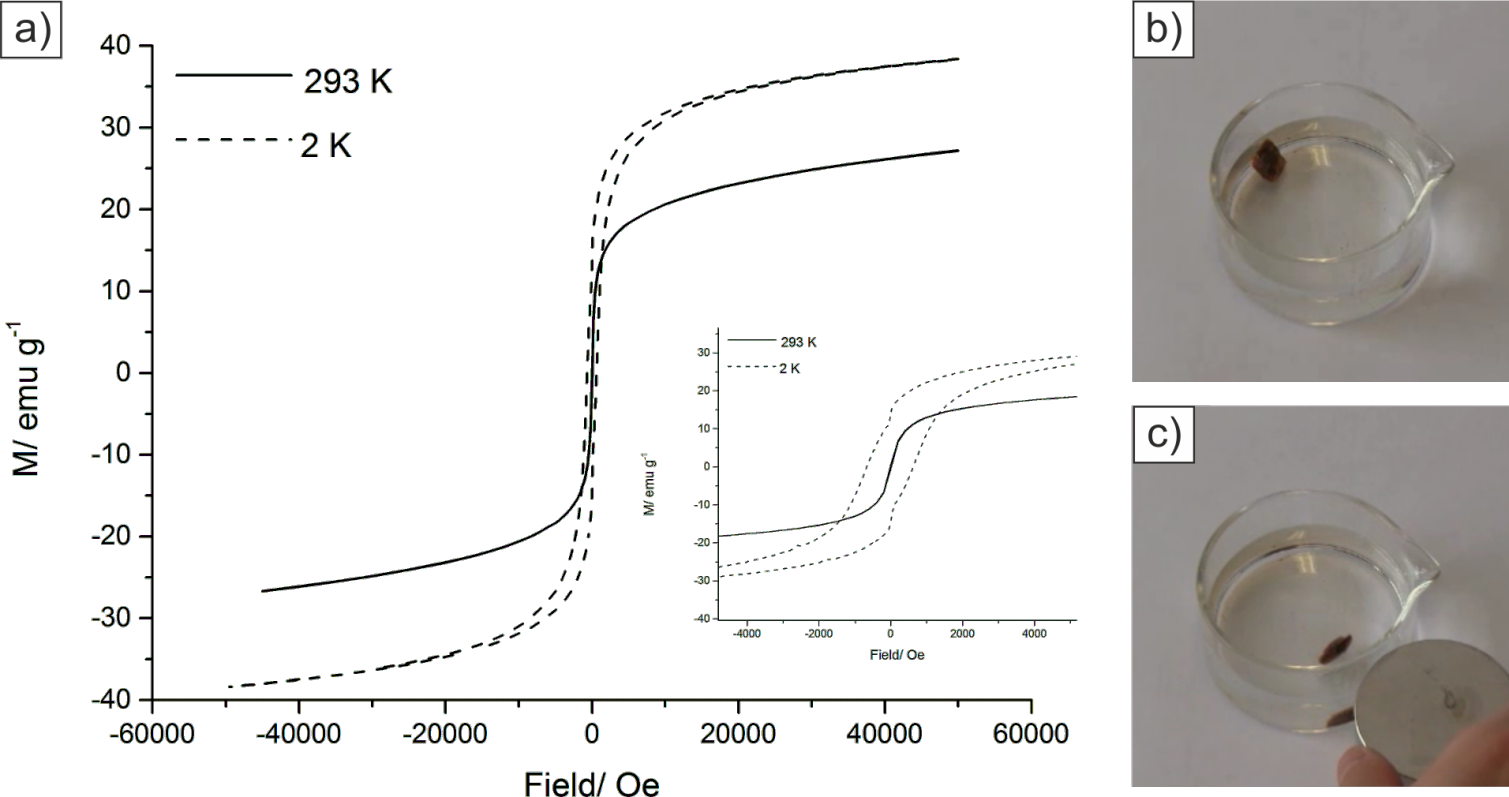
Magnetic properties of the synthesized hybrid materials. a) Magnetization curves of a representative dried sample at 2 K and 293 K. Inset: Enlargement of the low-field region showing the different coercive fields for the NPs at 2 and 293 K. Attraction of modified nacre with b) no magnetic field and c) external magnetic field (ca. 1 Tesla).

Magnetic properties of the nanocomposite were measured by using a superconducting quantum interference device (SQUID) magnetometer. Figure 7 illustrates the magnetization loops (magnetization *M* versus applied field *H*) of a representative dried hybrid material with a mineral content of 65 wt % after eight mineralization cycles at 293 K and 2 K. At *T* = 293 K the hysteresis curve shows zero coercivity and zero remanence as it is characteristic for superparamagnetic material [39] with a particle size less than 20 nm. Due to magnetic anisotropy the hysteresis curve at *T* = 2 K shows ferrimagnetic hysteresis. The saturation magnetization for all analyzed samples is around 26 emu/g at 298 K and 36 emu/g at 2 K which are similar values already reported before for the synthesis of gelatin-based magnetic hydrogels [34]. Similar results can be obtained for the analysis of magnetite nanoparticles prepared by a co-precipitation method in water [40–42]. In order to determine the effect of varying mineral content onto the magnetic properties, samples with a particle load of 15 wt % to 65 wt % have been analyzed. For all analyzed samples similar results for the magnetic hysteresis as well as for the saturation magnetization have been obtained. Therefore, we conclude that the mineral content as well as the transfer of the synthesis protocol to the layered organic matrix does not affect the magnetic nature of the material.

### Swelling studies

**Figure 8:**
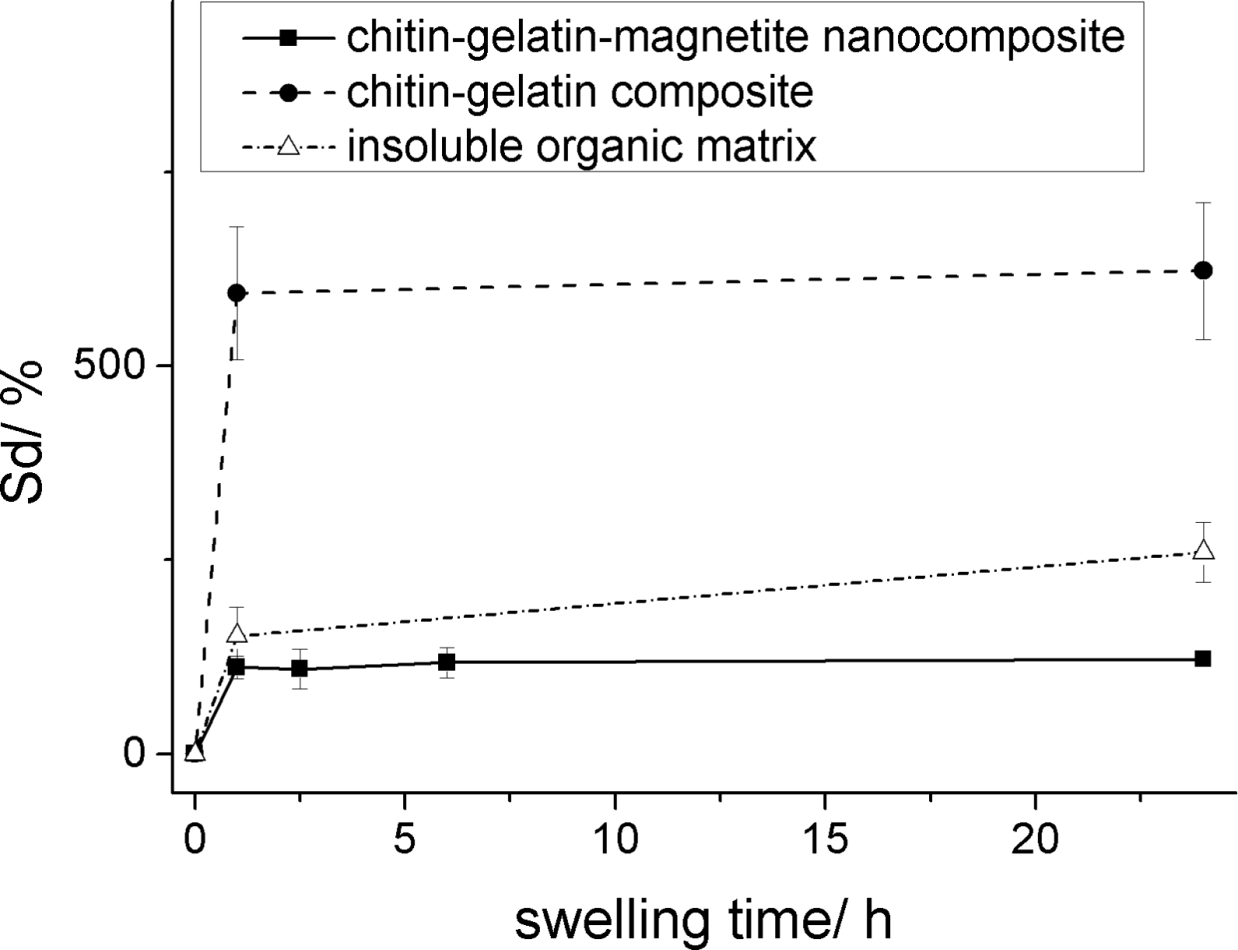
Degree of sample swelling plotted as a function of the swelling time at 23 °C for different samples with a gelatin concentration of 10 wt %. The equilibrium swelling degrees *Sd* (%) for the plotted samples are 622.97 ± 88.31 (chitin–gelatin), 259.70 ± 38.46 (chitin demineralized) and 121.94 ± 5.13 (chitin–gelatin–magnetite RC 6).

In order to probe structural changes of the nanocomposite during gelatin infiltration as well as during magnetite synthesis, swelling studies were performed. The swelling capacity of the insoluble nacre matrix, the gelatin infiltrated chitin matrix and the magnetic nanocomposite are shown in Figure 8. The swelling degree, *Sd*, is defined as:


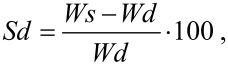


where *Ws* stands for the weight of the swollen sample after swelling equilibrium was reached and *Wd* stands for the dry weight before water uptake.

In the case of nacre matrix infiltrated with gelatin a distinct increase in swelling can be observed as compared to the insoluble matrix alone. This effect is not surprising as gelatin alone shows a higher swelling capacity as the insoluble organic matrix. The gravimetric water uptake of the gelatin–chitin composite is similar to already reported swelling capacities of gelatin. This observation is an additional proof for the successful infiltration of gelatin inside the chitin layers. In the case of the magnetic composite material, the swelling degree is significantly decreased due to the presence of magnetite nanoparticles, which act as additional crosslinkers in the gelatin hydrogel. This effect was discovered before for the studies of magnetic hydrogels [34] and shows similar values for the swelling degree. We can conclude that the gelatin hydrogel as well as the magnetic hydrogel do not change their swelling capacity inside the insoluble chitin matrix and therefore we conclude that the structural changes are similar than the one for already reported magnetic hydrogels.

### Simulation studies

To investigate the molecular scale interactions that account for the formation of the magnetite–protein composite, we performed molecular simulation studies of Fe^II^(OH)_2_ and Fe^III^(OH)_3_ motif association to two sets of biomolecular matrices. To allow direct comparison to our previous study on collagen-based composites [34,43], the association of an iron hydroxide ion cluster to collagen (mimicked by a triple helix of (Gly–Pro–Hyp)*_n_* peptides) is contrasted to ion association to chitin. The latter model was chosen as three poly-(1,4)-D-glucose chains of about 40 Å length (which corresponds to nine monomers) stacked in three layers, which are connected by hydrogen bonds.

**Figure 9:**
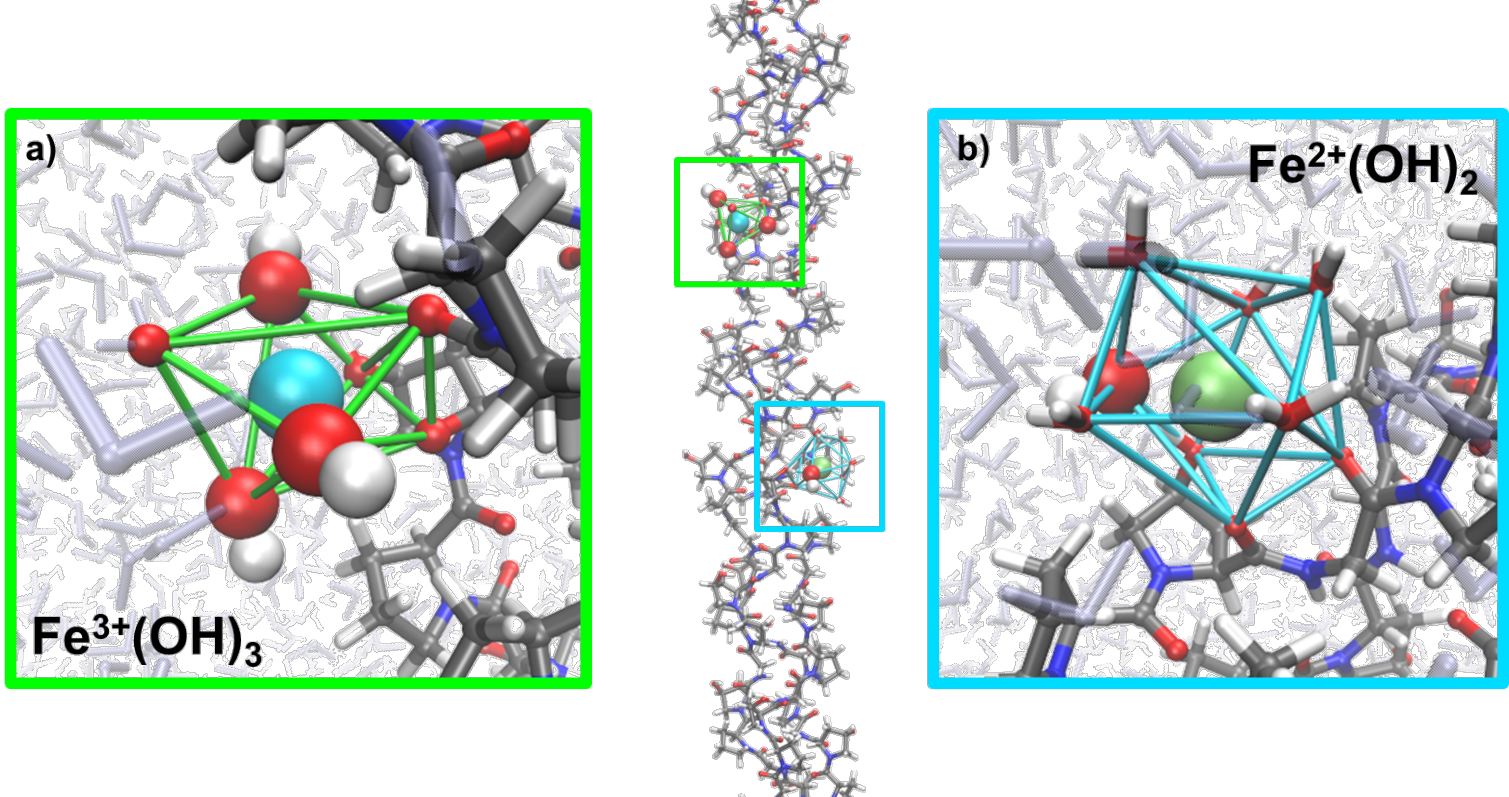
Representative structure of a triple helical (Gly–Hyp–Pro)*_n_* peptide [44] of 100 Å length with two associated iron clusters. a) The ferric ion (light blue) is coordinated by seven oxygen atoms of which the three hydroxides show the strongest interaction and an Fe–O distance of 2.7 Å. The Fe–O distances to the solvent and to carbonyl/hydroxy groups of collagen were found to be about 3 Å. b) The ferrous ion (green) is also coordinated by seven oxygen atoms, but does not show a bipyramidal structure. More importantly, one of the hydroxide ions dissociated into the solvent. The Fe–O distances for iron–collagen and iron–water contacts were found to be about 3 Å, whilst the remaining hydroxide ion exhibits an Fe–O distance of 3.2 Å. Colors: Fe^2+^ (green), Fe^3+^ (light blue), O (red), H (white), N (dark blue), C (grey).

**Figure 10:**
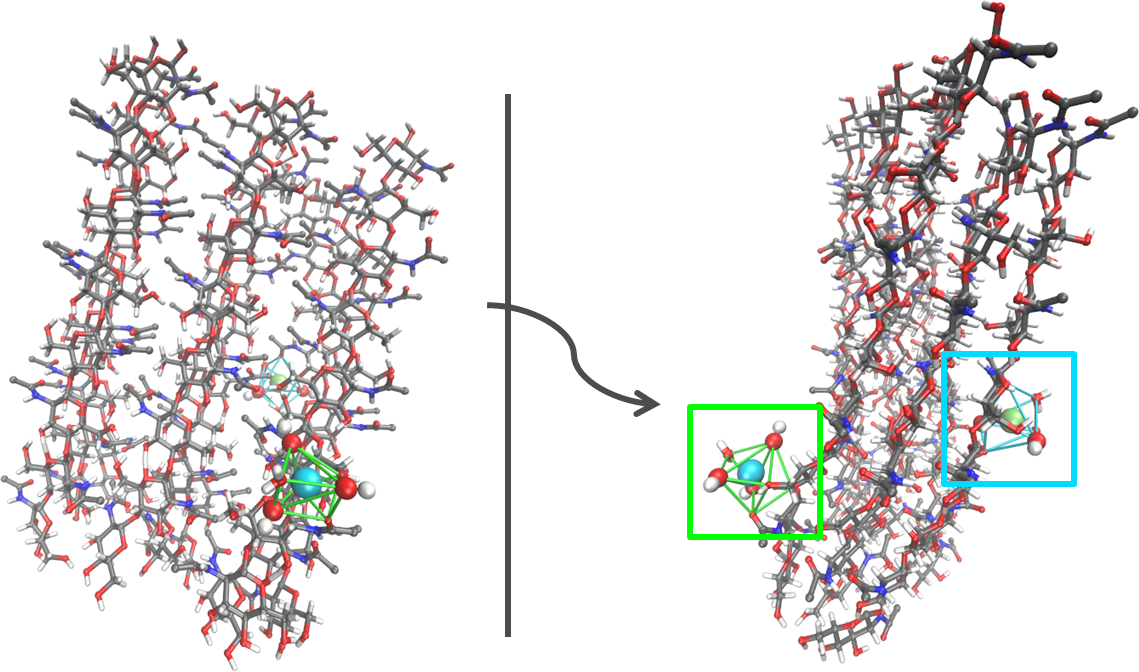
Illustration of a β-chitin model [45] consisting of three poly-(1,4)-D-glucose chains of nine monomers stacked in three layers.

**Figure 11:**
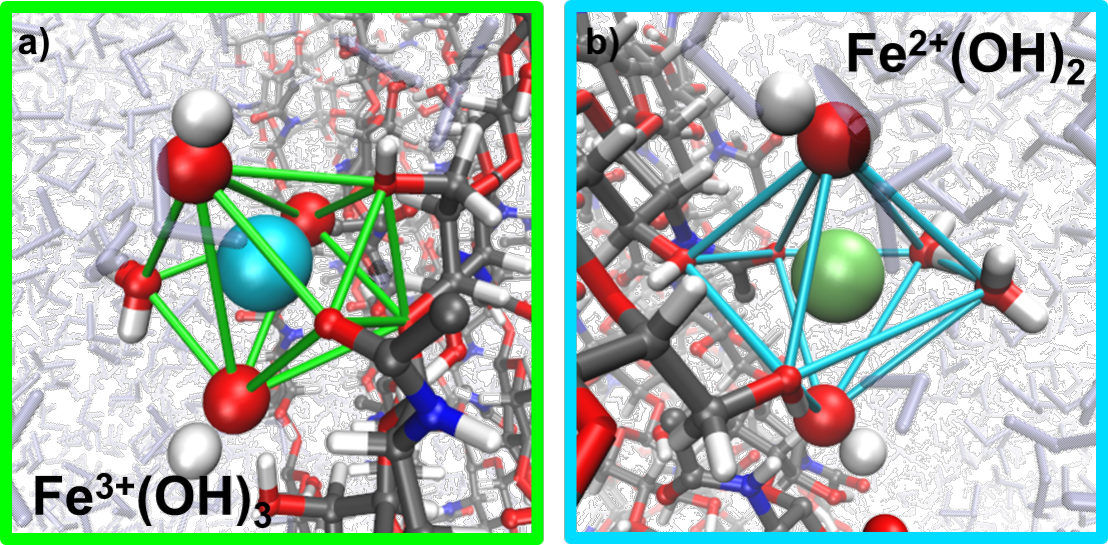
a) Representative structure for the coordination of Fe^III^(OH)_3_ by chitin. The ferric ion (light blue) is coordinated by four different types of oxygen atoms (red) forming seven coordinative interactions. b) Coordination of Fe^II^(OH)_2_ by chitin exhibiting a stable coordination by both hydroxide ions of the ion cluster. In summary, seven oxygen atoms coordinate the ferrous ion (green) building a pentagonal bipyramid, with the cluster hydroxide oxygens building the tops with a distance of 2.86 Å. The pentagonal plane consists of two oxygen atoms from solvent molecules forming weaker bonds of 3.1 Å and three protein contacts, whereby one carbonyl oxygen atom binds over 2.9 Å and two hydroxy oxygens over 3.1 Å.

As a starting point, the association of Fe^II^(OH)_2_ and Fe^III^(OH)_3_ ion clusters was investigated in vacuum. From a series of docking runs we found practically equivalent protein–ion complexes for either collagen or chitin. However, the nature of these complexes was found to differ significantly upon relaxation in aqueous solution. Figure 9 illustrates the association of the two ion cluster types to collagen and typical configurations as obtained from relaxation in aqueous solution based on 100 ps molecular dynamics simulation runs. While the Fe^III^(OH)_3_ ion clusters bind as stable moieties to the biomolecule, the association of Fe^II^(OH)_2_ to collagen was found to be less favored. Indeed, for 30% of the relaxation runs in aqueous solution the latter cluster was observed to partially dissociate, which led to (stable) Fe^II^(OH)^−^–collagen complexes. In contrast to this, the association of Fe^II^(OH)_2_ and Fe^III^(OH)_3_ ion clusters to chitin was found to be stable in both vacuum (Figure 10) and in aqueous solution (Figure 11). As the Fe^II^(OH)_2_ cluster reflects an important motif of the magnetite structure we conclude that our simulations show, at least from a qualitative point of view, a slight preference of chitin over collagen as a nucleator for magnetite [43].

### Mechanical characterization

**Figure 12:**
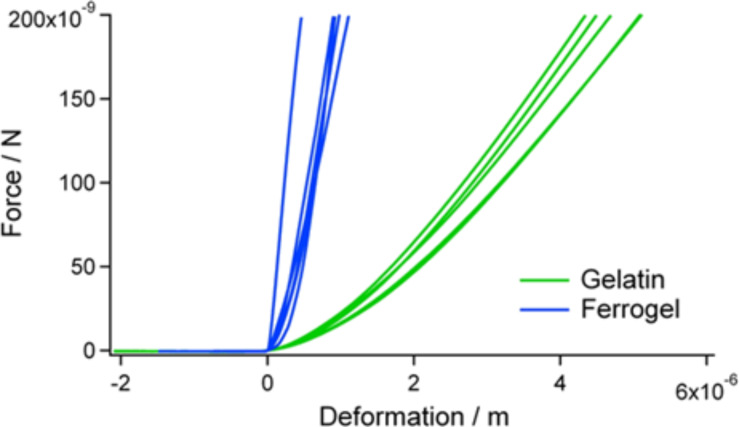
Force vs deformation characteristic of pure gelatin and gelatin with ferromagnetic particles. Introduction of nanoparticles leads to a significant increase of the stiffness of the material.

**Figure 13:**
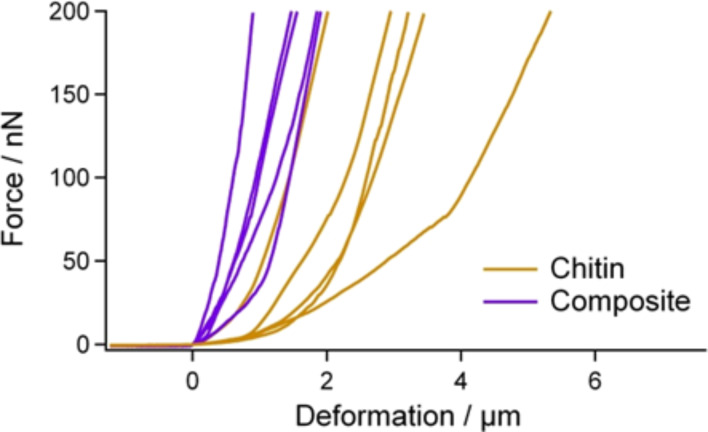
Force vs deformation characteristics of the chitin scaffold and the final composite. Introduction of ferrogel leads to a detectable increase of the stiffness of the material.

To examine the mechanical properties of the composite materials we conducted some preliminary experiments. Force spectroscopy measurements with the colloidal probe technique [46–47] were performed on bare and nanoparticle-loaded gelatin as well as on bare and ferrogel loaded chitin scaffolds. From the obtained force versus deformation curves we can already see significant qualitative differences. Figure 12 shows a comparison of pure and nanoparticle-filled gelatin. With the addition of the superparamagnetic particles the slope of the force curves increases, i.e., the stiffness or mechanical resistance of the gels is enhanced. This increase can be explained by the strengthening of the gelatin network by the rigid nanoparticles. These have been shown to interact with the amide bonds along the gelatin backbone [48] and might give rise to additional crosslinking. As a consequence, the flexibility of the gelatin chains is reduced resulting in the observed stiffness increase and the decreased swelling. Regarding the chitin scaffolds we notice a stiffening effect as well (Figure 13). Introducing the ferrogel reinforces the framework and gives the composite superior mechanical performance. Nanoindentation testing with AFM colloidal probe is a powerful technique as it combines high lateral and force resolution with well-defined contact geometry. It has successfully been applied to a range of systems including capsules [49–52], full particles [53–55] and films [56–59]. However, due to the morphological and structural inhomogeneity of our samples it is currently difficult to make a quantitative evaluation of the data. Continuum mechanics models typically require homogeneous and isotropic materials. For pure gelatin we can successfully fit the obtained curves assuming the Hertz model for a sphere in contact with a plane surface [60] (see Supporting Information File 1). Thus, an elastic modulus of 2.6 ± 0.3 kPa is calculated which is in good agreement with data from literature reporting modulus values in the low-kPa range [58,61]. In contrast, the data from experiments on ferrogel or composite show large scattering and the curves do not show a shape that can be described by one of the established mechanical theories. These deviations can be ascribed to the aforementioned non-ideal boundary conditions. It will be the aim of future research to investigate the mechanical properties more thoroughly.

## Conclusion

In summary, we have developed a synthetic method to fabricate a multifunctional hybrid material. We can successfully infiltrate gelatin into the insoluble nacre matrix and synthesize magnetite nanoparticles inside our template. We can control the mineral content of our hybrid material by repetition of reaction cycles, the mineral content varies form 15 wt % (one reaction cycle) to 65 wt % (eight reaction cycles). SQUID measurements showed that our composite material shows superparamagnetic behavior, which is typical for magnetite nanoparticles in this size range. Swelling studies indicate a structural change of the gelatin inside the hybrid material upon introduction of the magnetite nanoparticles. By incorporation of more and more inorganic material we can control the degree of swelling and therefore the mechanical properties of the composite material. This result is supported by preliminary AFM colloidal probe measurements. Simulation studies show the binding of iron and hydroxide ions to both collagen and β-chitin. Direct comparison, however, indicates that chitin should be the more favored nucleator macromolecule species for magnetite thus boosting composite growth along the chitin fibers.

In summary, we have managed to synthesize a bio-inspired organic–inorganic hybrid material, which combines the structural features of nacre and chiton teeth. Swelling studies and preliminary mechanical measurements indicate a change in the mechanical properties as compared to pure gelatin. This is controllable through the adjustable mineral content. In combination with the superparamagnetic behavior, we have therefore generated a material with improved mechanical performance coupled with magnetic properties. More quantitative future mechanical measurements will show in how far the fracture resistance of nacre could be combined with the wear resistance of chiton teeth.

## Experimental

### Chemicals

The following commercially available chemicals were purchased and applied in the syntheses without further purification: FeCl_2_·4H_2_O (Sigma-Aldrich), FeCl_3_·6H_2_O (Sigma-Aldich), 0.1 M NaOH solution (Merck), gelatin type B (~225 Bloom, Sigma-Aldrich), 4-chloro-*m*-cresol (Fluka), methanol (VWR). For the preparation of the reactant solutions double-distilled and deionized (Milli-Q) water was used. All solutions were degassed with argon before usage.

### Preparation of insoluble organic nacre matrix

Shells of *Haleotis laevigata* were sand-blasted to remove the calcite layer. After thorough washing with deionized water, the shells were dried overnight at room temperature and cut into pieces with an area of around 1 cm × 1 cm. The nacre pieces were demineralized with 10 vol % acetic acid and solvent exchange every day for at least 5 d. The remaining organic matrix was washed with Milli-Q water until neutral pH was reached.

### Gelatin preparation

The gelatin hydrogels were prepared as described elsewhere [34]. Here briefly, different amounts of gelatin powder were mixed with water and the gelatin granules were allowed to swell for 24 h at 6 °C. In order to obtain a homogeneous gel, the swollen mixture is heated for at least 2 h at 50 °C. 20 mL of the gelatin sol are filled into crystallization dishes and left at room temperature for gelation. In order to avoid bacterial growth, a 5 wt % solution of 4-chloro-*m*-cresol in methanol was added (0.15 mL per 1 g of gelatin granules).

### Infiltration of gelatin inside the insoluble nacre matrix

The cut demineralized insoluble organic nacre pieces are put into crystallization dishes filled with 20 mL liquid gelatin at 55 °C. To maintain uniform contact of the matrix pieces with the hot gelatin solution a filter paper covered the liquid surface to prevent floating. The complete set-up was then placed into a vacuum desiccator and the desiccator was attached to a vacuum pump. Vacuum was then applied until bubbling of the solution was observed. The vacuum was then removed to force the liquid gelatin to be drawn into the tissue. The whole process was repeated for three times. After gelatin infiltration the nacre matrix pieces were left inside the gelatin-filled crystallization dishes and allowed to stand for gelation first 5 h at room temperature and finally kept at 6 °C for 24 h before further usage. For further processing the gelatin-filled insoluble organic nacre parts were cut out of the gelatin hydrogel with a scalpel.

### Coomassie staining

Microtome cuts of embedded samples were incubated with 0.2 wt % Coomassie blue G-250 (Sigma-Aldrich) at room temperature for 2 h. After washing with acetic acid the cuts were carefully washed three times with destaining solution (30% ethanol/60% water/10% acetic acid).

### Rhodamine B ITC staining

Microtome cuts of embedded demineralized nacre matrix were incubated at 60 °C with 0.1 wt % rhodamine B ITC (Sigma-Aldrich) in water for 3 h. After washing with water the cuts were accurately washed with acidified ethanol for three times.

### In situ synthesis of magnetite nanoparticles

In situ mineralization of magnetite nanoparticles inside the gelatin hydrogel chitin composite material was carried out through co-precipitation of FeCl_2_ and FeCl_3_ after an already established synthesis protocol [34]. Briefly, the gelatin chitin composite sample was introduced into a solution, containing FeCl_2_ (0.1 M) and FeCl_3_ (0.2 M), where it was left for 96 h at 6 °C. The iron(II)- and iron(III)-loaded matrix was washed with water and placed in 0.1 M NaOH solution for 150 min.

### Sample characterizations

Samples of Coomassie-stained thin cuts were observed under bright field transmission mode by using a Zeiss optical microscope equipped with a video camera (AxioCam MRc5). Fluorescent labeled samples were analyzed with a confocal fluorescence laser scanning microscope (Zeiss LSM 510 Meta) at an excitation wavelength of 543 nm.

For TEM examination the formed composite material was dehydrated with a graded ethanol series and embedded in LR White Resin (Medium Grade). The sample was cut perpendicular with a diamond knife in a Leica ultracut UCT and transferred onto a Formvar-coated copper grid. TEM and electron diffraction were performed on a Zeiss Libra 120 operating at 120 kV. For SEM measurements the samples were air-dried at room temperature and cut perpendicular to the chitin layers with a scalpel. The sample was placed on a sticky carbon tape and coated with a thin layer of gold in order to avoid charging effects. The SEM measurements were performed on Zeiss Neon 40 EsB operating in high vacuum. An InLens and SE detector was used for signal collection and an acceleration voltage of 5 kV was chosen for recording the images.

Small-angle neutron scattering (SANS and VSANS): SANS and VSANS experiments were carried out at the KWS1 and KWS 3 diffractometers operated by Jülich Center for Neutron Research (JCNS) at the Forschungs-Neutronenquelle Heinz Maier-Leibnitz (FRM II) in Garching, Germany [62]. Some of the SANS data at large Q range is based on experiments performed at the SANS II, Swiss spallation neutron source SINQ, Paul Scherrer Institute, Villigen, Switzerland.

The mineral content of the multifunctional hybrid material was determined by means of TGA (Netzsch, Selb, Germany). Measurements were carried out at a heating rate of 5 K/min under a constant oxygen flow. Samples were scanned from 293 K to 1273 K.

Magnetization measurements were carried out by using a quantum design superconducting quantum interference device (SQUID) 5 T magnetic properties measurement system (MPMS). For measurements, dried samples were introduced into gelatin capsules and magnetization loop measurements at 2 K and 293 K were performed.

### Simulation studies

Molecular Simulation: as described in [34] a series of Fe^III^(OH)*_x_*(OH_2_)_4−_*_x_* and Fe^II^(OH)*_y_*(OH_2_)_6−_*_y_* clusters were pre-modeled from ab-initio calculations in vacuum. For all clusters high-spin constellation was identified as preferred by several electron volts. Imposing overall charge neutrality (i.e., *x* + *y* = 3 + 2) we found the neutral Fe^III^(OH)_3_·(H_2_O) and the Fe^II^(OH)_2_·4(H_2_O) as energetically preferred. Docking to collagen and chitin was modeled in aqueous solution by using empirical force fields [44–45,63–64]. Investigation of biologically designed metal-specific chelators for potential metal recovery and waste remediation applications [65], and the Kawska–Zahn docking procedure were described previously [43].

Along this line, ion clusters initially docked to collagen/chitin in absence of water. Such putative association complexes are then immersed in aqueous solution (periodic simulation cell comprising more than 15000 water molecules) and subjected to relaxation from 100 ps molecular dynamics runs at room temperature and ambient pressure. To account for the manifold of possible arrangements intrinsic to the systems complexity a series of 200 independent docking runs were performed for each ionic species.

### Mechanical characterization

Force spectroscopy experiments were conducted at the atomic force microscope (AFM) Nanowizard^®^ I (JPK Instruments, Berlin, Germany) in a custom-built liquid cell (diameter 2 cm, height 0.5 cm). Thin slices (1–2 mm) of swollen hydrogels were cut from the bulky samples with a scalpel and immobilized at the bottom of the cell by using two component epoxy glue (UHU Endfest 300, UHU GmbH & Co. KG, Bühl, Germany). All measurements were performed in Milli-Q-water at room temperature. As a probe a tipless silicon nitride cantilever (NSC 12, no Al coating, MikroMasch, Tallinn, Estonia) was used with a glass sphere (35 µm in diameter, Polysciences Europe GmbH, Eppelheim, Germany) attached to its front (colloidal probe). Before the actual measurements, the cantilevers were calibrated against the non-deformable glass substrate to determine their optical lever sensitivity resulting as the slope of the recorded force–displacement curve. The deformation of the sample was obtained by subtraction of the bending of the cantilever from the raw displacement data. The spring constant of the cantilever (0.56 N/m) was deduced from its thermal noise spectrum prior to the attachment of the colloidal probe [66].

## Supporting Information

File 1 Additional experimental data.
