# Supplementary material for: Multifunctional layered magnetic composites
Source: Beilstein J Nanotechnol. 2015 Jan 12;6:134–48. doi: 10.3762/bjnano.6.13 (PMC4311584; doi:10.3762/bjnano.6.13)
Supplement: File 1 — Additional experimental data. [file Beilstein_J_Nanotechnol-06-134-s001.pdf]

Supporting Information

for

Multifunctional layered magnetic composites

Maria Siglreitmeier[1], Baohu Wu[1,2], Tina Kollmann[3], Martin Neubauer[4], Gergely Nagy[5], Dietmar Schwahn[6], Vitaliy Pipich[2], Damien Faivre[7], Dirk Zahn[3], Andreas Fery[4] and Helmut Cölfen*[1]

Address: [1]Department of Chemistry, Physical Chemistry, University of Konstanz, Universitätsstraße 10, 78457 Konstanz, Germany, [2]Jülich Centre for Neutron Science JCNS-MLZ, Outstation at MLZ, Forschungszentrum Jülich, Lichtenbergstraße 1, 85748 Garching, Germany, [3]Theoretical Chemistry, University of Erlangen-Nürnberg, Nägelsbachstraße 25, 91052 Erlangen, Germany, [4]Physical Chemistry II, University of Bayreuth, Universitätsstraße 30, 95447 Bayreuth, Germany, [5]Laboratory for Neutron Scattering, Paul Scherrer Institute, 5232 Villigen PSI, Switzerland, [6]Technische Universität München, Forschungs-Neutronenquelle Heinz Maier-Leibnitz (FRM II), 85748 Garching, Germany and [7]Department of Biomaterials, Max Planck Institute of Colloids & Interfaces Science Park Golm, 14424 Potsdam, Germany

Email: Helmut Cölfen - helmut.coelfen@uni-konstanz.de

* Corresponding author

Additional experimental data

***Small-Angle Neutron Scattering (SANS and VSANS)**:* As described in [1], SANS and VSANS experiments were carried out at the KWS1 and KWS 3 diffractometers operated by by Jülich Center for Neutron Research (JCNS) at the Forschungs-Neutronenquelle Heinz Maier-Leibnitz (FRM II) Garching, Germany [2]. Three configurations were used at KWS 1, namely the sample-to-detector (SD) distances of 2, 8 and 20 m and the corresponding collimation lengths of 8 and 20 m. The wavelength was 0.7 nm ($\Delta\lambda/\lambda$ = 10%). These settings allowed covering a *Q*-range from 0.02 to 3.5 nm^{-1}. The scattering vector *Q* is defined as $Q = \frac{4\pi}{\lambda}\sin(\theta/2)$ with the scattering angle θ and the wavelength λ. A two-dimensional local sensitive detector detected the neutrons scattered by the sample solutions. The samples were fixed in a sandwich quartz cell with a path-length of about 1 mm. Plexiglas was used as secondary standard to calibrate the scattering intensity in absolute units. The data correction and calibration were performed using the software described in [3]. Some of the SANS data were measured at SANS II at Paul Scherrer Institute (PSI) in Villigen, Switzerland. The sample-to-detector distances were 1 and 5 m, the corresponding collimation lengths 4 and 5 m, and the wavelength 0.52 nm. These settings allowed us to cover a *Q*-range from 0.1 to 3.5 nm^{-1}.

In order to become sensitive to larger length scales of the order of micrometers, i.e., the network structure of the solutions, we extended our research at the very-small-angle neutron scattering (VSANS) diffractometer KWS3. This instrument uses a parabolic mirror as a focusing optical element and covers a *Q*-range from 0.001 to 0.02 nm^{-1}.

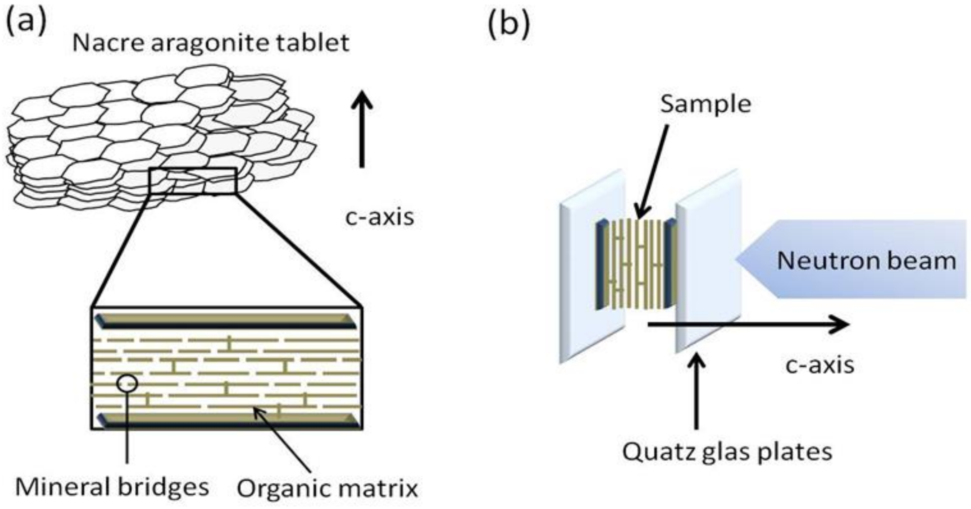

Figure S1: (a) the nacre tablet structure and (b) SANS scattering geometry on the nacre and nacre organic matrix.

Small-angle X-ray scattering (SAXS:) SAXS experiments were carried out similarly as described in [1] at a HECUS S3-Micro small-angle X-ray scattering instrument. The instrument uses Cu Kα radiation (0.154 nm) produced in a sealed tube. Samples were placed in Hilgenberg quartz capillaries with an outside diameter of 1 mm and wall thickness of 0.01 mm. The scattered intensity was corrected with the transmission of the samples calculated considering the absorption of the sample and that of the capillary. The scattered X-rays are detected with a two-dimensional multiwire area detector and afterwards converted to one-dimensional scattering by radial averaging and represented as a function of momentum transfer vector Q similar to the SANS experiments.

The Beaucage expression

The Beaucage expression is given according to

$$\frac{d\Sigma}{d\Omega}(Q) = \frac{d\Sigma}{d\Omega}(0)\ \exp(-u^2/3) + P_\alpha \left[\left(\mathrm{erf}\left(u/\sqrt{6}\right)\right)^3 /Q\right]^\alpha \qquad \text{(S1)}$$

representing a combination of Guinier's and Porod's laws describing the scattering at low and large *Q*, respectively. More quantitatively both approximations are valid for the parameter $u = R_gQ$ smaller or larger than 1, with *u* representing the product of radius of gyration R_g and scattering vector *Q* (defined below). Guinier's law has the shape of a Gaussian function whereas for *Q* larger than $1/R_g$ ($u > 1$) a power law according to $d\Sigma / d\Omega(Q) = P_\alpha Q^{-\alpha}$ is often observed, which in case of $\alpha = 4$ represents the famous Porod law of compact particles with a sharp surface [4].

The correlation model:

$$\frac{d\Sigma}{d\Omega}(Q) = \frac{A}{Q^n} + \frac{C}{1+(Q\xi)^m} + \mathrm{BKG} \qquad \text{(S2)}$$

In the above equation, the first term describes Porod scattering from clusters (exponent = *n*) and the second term is a Lorentzian function describing scattering from polymer chains (exponent = *m*). This second term characterizes the polymer/solvent interactions and therefore the thermodynamics. The parameter (ξ) is a correlation length for the polymer chains. This correlation length represents a weighted-average inter distance between the hydrogen/deuterium-containing groups. The multiplicative factors of the Porod and Lorentzian terms (A and C, respectively), the Q independent incoherent background scattering (BKG), and the lower-Q and higher-Q scattering exponents (*n* and *m*, respectively) were obtained by a nonlinear, least squares fit of the data.

Gelatin

Gelatin is derived from partial hydrolysis of native collagen and can be considered as a polydisperse copolymer with a broad molar mass distribution. At temperatures above the gelation temperature (T_{gel}) gelatin and below the overlap concentration of about 0.5 wt % (in H_2O) native collagen forms a homogeneous solution in water. This is seen from the SAXS data in Figure S2. It is seen that the biopolymer of 0.3 wt % concentration has a correlation length of ξ = 15.9 ± 0.5 nm and shows the characteristics of swollen Gaussian chains in a good solvent. The latter characterization follows from the α = 1.6 power law exponent which corresponds to the Flory exponent $\nu = 3/5$ ($\nu \equiv 1/\alpha$) [5]**.** The correlation length of ξ is smaller than the typical mineral bridge size of the nacre insoluble matrix (ca. 70 nm) and indicates that the gelatin molecular diffusion into the organic matrix of demineralized nacre through the holes of former mineral bridges is possible.

Below T_{gel} and above the overlap concentration of ca. 0.5 wt % (in H_2O) gelatin transforms to a thermoreversible physical gel, which is crosslinked by the association of helical domains. Most of the chains form a three-dimensional interconnected network transforming the random coil as observed in Figure S2 to a triple-helical structure. According to our previous SANS studies [1] the gel structure depends on the concentration. The choice of appropriate gelatin concentration will achieve both high efficiency for ion transport for optimal iron mineralization and high mechanical strength.

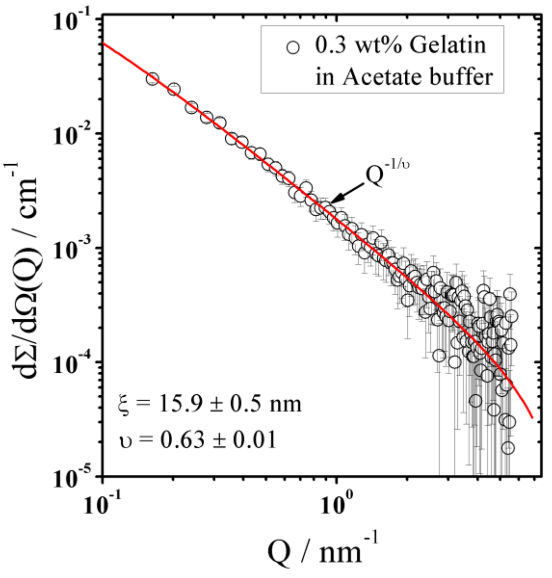

Figure S2: SAXS macroscopic cross-section dΣ/dΩ versus scattering vector Q for gelatin in H_2O with acetate buffer (T = 20 °C, pH 5.3). The solid line represents a fit of correlation model [6]. The exponent ν = 0.63 corresponds to the "Flory" exponent of a swollen linear chain in three dimensions according to ν = 3/5 [5].

Light microscopy studies

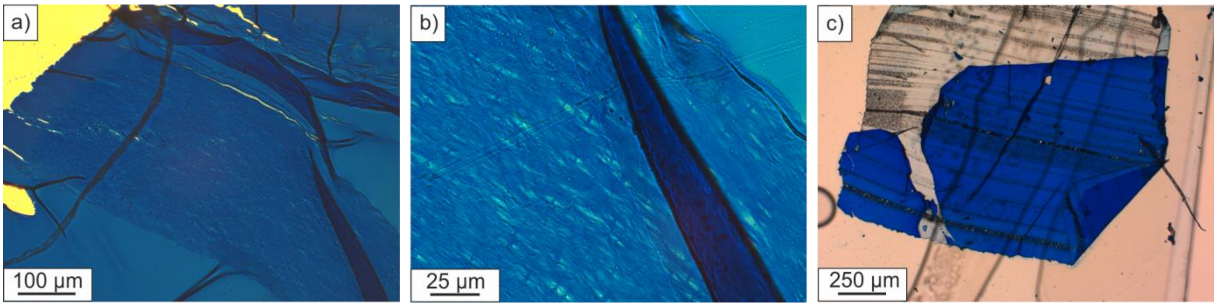

Figure S3: Light microscopy image of thin cut of embedded and Coomassie stained samples a) and b) demineralized nacre matrix with infiltrated gelatin without any digital modification c) reference experiment of embedded and microtomed gelatin stained with Coomassie blue.

TGA measurements

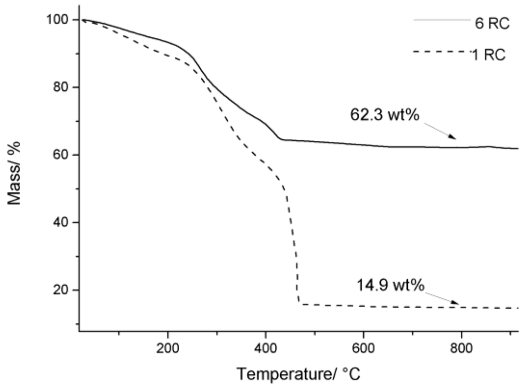

Figure S4: TGA curves of chitin–gelatin–magnetite (10 wt % gelatin) composite after different reaction cycles. RC stands for the number of reaction cycles.

Diffraction analysis

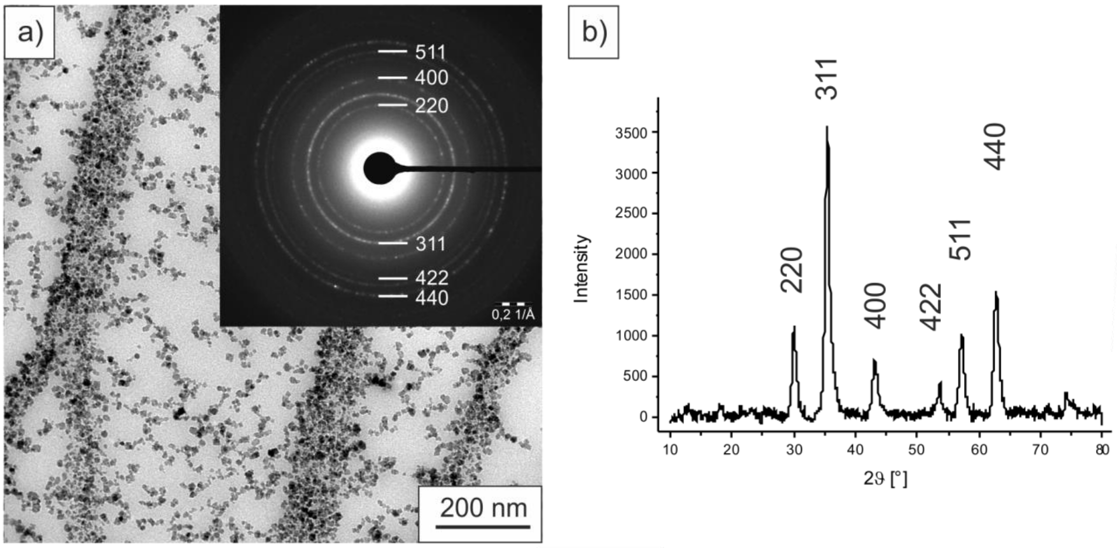

Figure S5: a) TEM image of ultramicro-cut of embedded gelatin-chitin-magnetite composite with selected area electron diffraction (SAED) image. B) XRD pattern of the representative magnetic composite material.

Mechanical characterization

The Hertz model is one of the fundamental theories to describe the elastic deformation of two bodies in contact [7]. It assumes homogeneous, isotropic and linear elastic bodies with negligible adhesion or friction during deformation. For normal loading and small deformations (10 % of film thickness or particle diameter) the applied force *F* scales with deformation *d* like:

$$F = \frac{4}{3}\, E\, d^{\frac{3}{2}} \sqrt{R} \qquad \text{(S3)}$$

Here, *E* is the relative Young's modulus and *R* is the radius of a sphere (in our case the colloidal probe). The relative modulus is composed of the moduli E_i and Poisson's ratio v_i of the two bodies in contact:

$$\frac{1}{E} = \frac{1-{\nu_1}^2}{E_1} + \frac{1-{\nu_2}^2}{E_2} \qquad \text{(S4)}$$

From the above Equation 1 it follows that plotting the logarithm of force vs deformation the slope should equal 1.5 which can be taken as a simple check for the validity of the model.

The examined pure gelatin samples could be fitted very well with this model (fit error less than 1%) and the slope of the log–log plots matches the 1.5 predicted by the Hertz model.

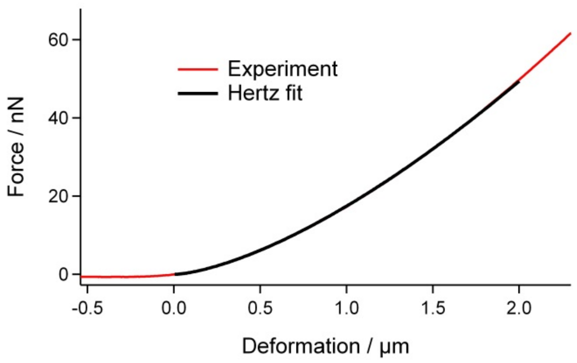

Figure S6: Exemplary force–deformation characteristic on pure gelatin (14 wt %) and corresponding Hertz fit.

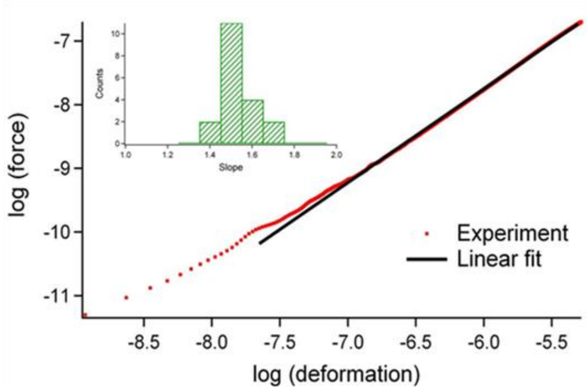

Figure S7: Logarithmic representation of the force–deformation curve from Figure S6 with linear fit indicating a slope of 1.47, close to the predicted 1.5 from Hertzian theory. Inset: statistic evaluation of log–log slopes (19 curves), mean value = 1.49 ± 0.08.

References

1. Helminger, M.; Wu, B.; Kollmann, T.; Benke, D.; Schwahn, D.; Pipich, V.; Faivre, D.; Zahn, D.; Cölfen, H., *Adv. Funct. Mater.* **2014,** *24,* 3187–3196.
2. Home – MLZ – Heinz Maier-Leibnitz Zentrum. http://www.mlz-garching.de/englisch (accessed, December, 9, 2013).
3. [Vitaliy Pipich] qtikws. http://www.qtikws.de (accessed, December, 9, 2013).
4. Roe, R. J. *Methods of X-ray and Neutron Scattering in Polymer Science;* Oxford University Press: New York, 2000.
5. Rubinstein, M.; Colby, R. H. *Polymer Physics;* Oxford University Press, 2006; p 104.
6. Hammouda, B.; Ho, D. L.; Kline, S. *Macromolecules* **2004,** *37,* 6932–6937.
7. Hertz, H. *J. Reine Angew. Math.* **1881,** *92*, 156—171.
